# Intraoperative hyperspectral label-free imaging: from system design to first-in-patient translation

**DOI:** 10.1088/1361-6463/abfbf6

**Published:** 2021-05-14

**Authors:** Michael Ebner, Eli Nabavi, Jonathan Shapey, Yijing Xie, Florentin Liebmann, José Miguel Spirig, Armando Hoch, Mazda Farshad, Shakeel R Saeed, Robert Bradford, Iain Yardley, Sébastien Ourselin, A David Edwards, Philipp Führnstahl, Tom Vercauteren

**Affiliations:** 1 School of Biomedical Engineering & Imaging Sciences, King’s College London, London, United Kingdom; 2 Wellcome / EPSRC Centre for Interventional and Surgical Sciences, UCL, London, United Kingdom; 3 Department of Neurosurgery, National Hospital for Neurology and Neurosurgery, Queen Square, London, United Kingdom; 4 Research in Orthopedic Computer Science (ROCS), Balgrist University Hospital, University of Zurich, Balgrist CAMPUS, Zurich, Switzerland; 5 Laboratory for Orthopaedic Biomechanics, ETH Zurich, Zurich, Switzerland; 6 Department of Orthopaedics, Balgrist University Hospital, University of Zurich, Zurich, Switzerland; 7 The Ear Institute, UCL, London, United Kingdom; 8 The Royal National Throat, Nose and Ear Hospital, London, United Kingdom; 9 Department of Paediatric Surgery, Evelina London Children’s Hospital, London, United Kingdom

**Keywords:** hyperspectral imaging, hyperspectral imaging, medical device, translational research, computer assisted interventions, exoscope, first-in-patient

## Abstract

Despite advances in intraoperative surgical imaging, reliable discrimination of critical tissue during surgery remains challenging. As a result, decisions with potentially life-changing consequences for patients are still based on the surgeon’s subjective visual assessment. Hyperspectral imaging (HSI) provides a promising solution for objective intraoperative tissue characterisation, with the advantages of being non-contact, non-ionising and non-invasive. However, while its potential to aid surgical decision-making has been investigated for a range of applications, to date no real-time intraoperative HSI (iHSI) system has been presented that follows critical design considerations to ensure a satisfactory integration into the surgical workflow. By establishing functional and technical requirements of an intraoperative system for surgery, we present an iHSI system design that allows for real-time wide-field HSI and responsive surgical guidance in a highly constrained operating theatre. Two systems exploiting state-of-the-art industrial HSI cameras, respectively using linescan and snapshot imaging technology, were designed and investigated by performing assessments against established design criteria and *ex vivo* tissue experiments. Finally, we report the use of our real-time iHSI system in a clinical feasibility case study as part of a spinal fusion surgery. Our results demonstrate seamless integration into existing surgical workflows.

## 1. Introduction

Many difficult intraoperative decisions with potentially life-changing consequences for the patient are still based on the surgeon’s subjective visual assessment. This is partly because, even with the most advanced current surgical techniques, it may still not be possible to reliably identify critical structures during surgery. Neuro-oncology and orthopaedic surgery are among the specialties that benefited most from advanced visualisation techniques and computer-assisted technologies. Navigation solutions have for example been presented for brain (Gerard *et al* 2017) and spinal (Helm *et al* 2015) procedures to map preoperative information such as magnetic resonance imaging (MRI) or computed tomography (CT) to the anatomy of the patient on the surgical table. However, navigation based on preoperative imaging cannot reliably account for intraoperative changes creating uncertainty for surgical decision making. Interventional imaging and sensing, such as surgical microscopy, fluorescence imaging, point-based Raman spectroscopy, ultrasound and intra-operative MRI, may be used by the surgeon either independently or as adjunct to navigation information to visualise the operated tissues. However, tissue differentiation based on existing intraoperative imaging remains challenging because of stringent operative constraints in the clinical environment (e.g. intraoperative MRI or CT), or imprecise tumour delineation (e.g. ultrasound).

Advanced optical imaging techniques provide a promising solution for intraoperative tissue characterisation, with the advantages of being non-contact, non-ionising and non-invasive. By splitting light into multiple narrow spectral bands far beyond what the naked eye can see, hyperspectral imaging^11^ 11Depending on the number of acquired bands, hyperspectral imaging may also be called *multispectral imaging*. We will continue to refer to hyperspectral imaging regardless of the number of bands used for simplicity. (HSI) carries diagnostic information about tissue properties that can be used for objective tissue characterisation without the need of any exogenous contrast agent.

As a label-free imaging modality, HSI and its diagnostic capabilities have been explored for biomedical imaging applications over many years (Lu and Fei 2014, Hu *et al* 2017, Halicek *et al* 2019, Shapey *et al* 2019, Clancy *et al* 2020) In particular, it has been demonstrated that the spectral signature of tissues captured by HSI can provide both quantitative functional (e.g. blood perfusion and oxygenation saturation levels) (Klaessens *et al* 2013, Mori *et al* 2014) and semantic (e.g. tissue type such as tumour vs healthy) (Kho *et al* 2019, Fabelo *et al* 2018) tissue information that are particularly interesting for surgical decision making. However, whilst HSI has been investigated for the assessment of various clinical conditions such as peripheral vascular disease (Chiang *et al* 2017), retinal eye disease (Desjardins *et al* 2016), hemorrhagic shock (Cancio *et al* 2006), healing in foot ulcers of diabetic patients (Khaodhiar *et al* 2007) and cancer detection (Fei *et al* 2017), its *in vivo* surgical use has been restricted to a few clinical research cases only (Shapey *et al* 2019). For example, while the HELICoiD research system (Fabelo *et al* 2018) demonstrated promising clinical research results for *in vivo* brain tumour detection (Fabelo *et al* 2019), its size is prohibitive for clinical adoption during surgery. Other systems presented for the intraoperative assessment of tissue perfusion and oxygenation—including breast (Gioux *et al* 2011), oral cancer (Klaessens *et al* 2013), renal (Best *et al* 2013), epilepsy (Noordmans *et al* 2013), neurovascular (Mori *et al* 2014) and gastrointestinal surgery (Yoon *et al* 2019, Barberio *et al* 2020)—further demonstrate the potential of iHSI. Yet, these are prone to produce motion artefacts due to insufficient imaging speed for a dynamic scene during surgery. More recently, two intraoperative systems based on pushbroom HSI cameras were presented that allow for integration into the surgical workflow: In Mühle *et al* (2021), the TIVITA system (Kulcke *et al* 2018) was attached to a surgical microscope to capture *in vivo* neurosurgery data; in Köhler *et al* (2020), a laparoscopic HSI camera was presented and tested using resected esophagus tissue and in Hu *et al* (2020) a HSI imaging system was tested during liver cancer surgery. While these systems show potential for seamless integration into the surgical workflow, their restricted imaging speed is likely to remain an inhibitor for adoption during surgery. For increased real-time imaging speed, recently developed *snapshot* HSI camera systems have been used to assess brain perfusion in neurosurgery (Pichette *et al* 2016) and to perform preclinical skin perfusion analysis (Ewerlöf *et al* 2017). However, while snapshot HSI sensors permit real-time HSI capture with video-rate imaging, spatial resolution is limited and needs to be accounted for in a post-processing step called *demosaicking* (Dijkstra *et al* 2019, Tsagkatakis *et al* 2019). Moreover, previously presented snapshot iHSI works did not methodologically map out and address the critical design considerations to ensure a seamless integration into the surgical workflow.

While various HSI systems have been tested in a surgical environment to investigate the potential of iHSI, to the best of our knowledge, no HSI system has been presented allowing for strict clinical requirements including a means of maintaining sterility and ensuring seamless integration into the surgical workflow that can provide real-time information for intraoperative surgical guidance.

**Figure 1.**
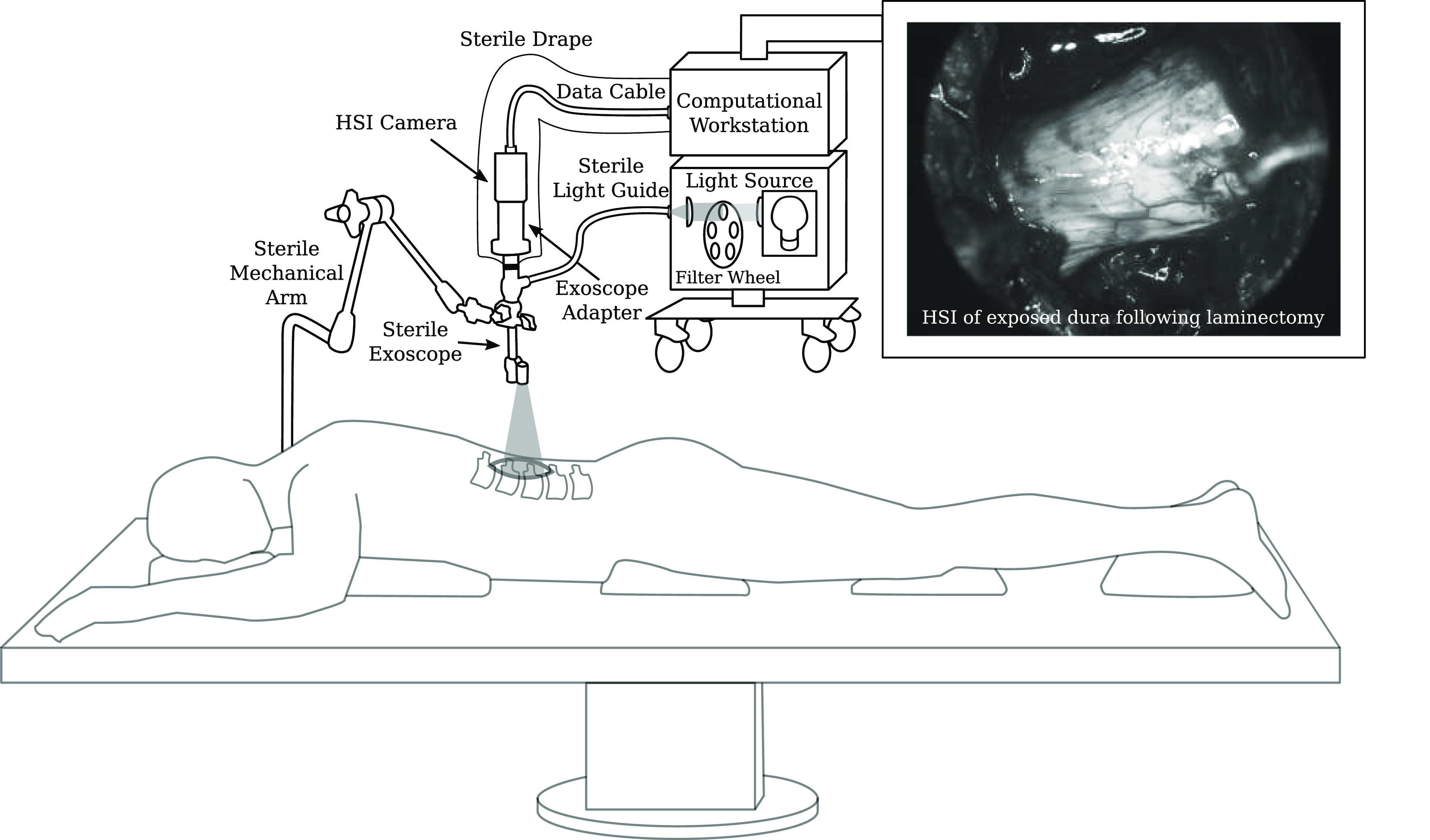
Schematic diagram of our intraoperative hyperspectral imaging (HSI) system illustrated for the example of spine surgery. A snapshot HSI camera system was used for the in-patient clinical feasibility case study as part of a spinal fusion surgery. Video-rate HSI data was acquired during surgery. An example *in vivo* snapshot hyperspectral mosaic image demonstrating the exposed dura of the spinal cord following laminectomy is provided.

In this paper, our contributions are four-fold: (a) by building on our preliminary work (Shapey *et al* 2018), we first present a set of design requirements, including functional and technical requirements, critical for an iHSI system to provide real-time wide-field HSI information for seamless surgical guidance in a highly constrained operating room (OR); (b) we present and evaluate our developed iHSI system against these requirements by considering two state-of-the-art industrial HSI camera systems based on *linescan* and *snapshot* imaging technology as further described in section 3.1; (c) we perform *ex vivo* animal tissue experiments in a controlled environment with our proposed iHSI setup to investigate tissue properties using both camera systems; and (d) we report the use of our real-time iHSI system (figure 1) during an ethically-approved in-patient clinical feasibility case study as part of a spinal fusion surgery therefore successfully validating our assumptions that this system can be seamlessly integrated into the OR without interrupting the surgical workflow.

## 2. Intraoperative HSI system for real-time surgical guidance

In this section, we present the key design requirements of an HSI for intraoperative surgical guidance suitable for open surgery. By following these criteria, the iHSI system illustrated in figure 1 is introduced and described.

### 2.1. Intraoperative HSI system design requirements

Our main design assumption is that the intraoperative application of an HSI camera system is facilitated by developing a standalone light-weight device independent of an operating microscope typically used for neurosurgery. In particular, by ensuring compatibility with surgical *telescopes*, such as an exoscope (Ricciardi *et al* 2019) or endoscope, a modular and flexible system design can be achieved suitable for both open or endoscopic surgery across surgical specialities. Following this assumption, tables 1 and 2 provide an overview of design requirements considered for a hyperspectral imaging system for intraoperative surgical guidance including minimum and target requirements. These are divided into (a) functional requirements, i.e. requirements imposed by the clinical environment in the OR during surgery (table 1), and (b) technical requirements, i.e specifications for a HSI system to achieve high-fidelity imaging data to satisfy the listed functional requirements for the purpose of real-time surgical guidance (table 2). When objective requirements cannot be provided, best estimates are given based on our experience as outlined below.

**Table 1.** Overview of functional design requirements of a hyperspectral imaging system for intraoperative surgical guidance. Corresponding technical requirements from table 2 are listed in the rightmost column.

	Minimum Requirement	Target Requirement	Req
**F1** Surgical safety and sterility	Safe and sterile intraoperative use must be possible throughout the surgical procedure.	Ibid.	T1,T2,T8,T9,T12
**F2** Technical safety	Device must comply with electrical and light source safety standards so that it may be used safely within the operating theatre without causing tissue injury.	Ibid.	T1,T2,T4–T6,T8,T9,T16,
**F3** Lighting	Light and illumination requirements must not impede surgical workflow.	Ibid. Additionally, light and illumination can be adjusted to accommodate the surgeon’s needs.	T7,T10,T18
**F4** Maintenance	Maintenance and cleaning requirements must comply with standard clinical practice.	Ibid.	T1,T2
**F5** Device handling	Device must be securely held or mounted during the procedure but easily manoeuvrable.	Handheld device must be easily manoeuvrable and be light enough to position securely without the need for an assistant.	T2–T6,T10,T12
**F6** Anatomical coverage	Field of view (FOV) and depth of imaging must provide information compatible with the surgical action.	Ibid. Additional monitoring capabilities are available.	T11–T13,T19
**F7** Anatomical feature	Critical functional or semantic features to increase surgical precision and patient safety during the procedure.	Multiple functional and semantic features to increase surgical precision and patient safety for comprehensive patient monitoring.	T7,T11 and T13–T19
**F8** Anatomical detail	Resolution suitable to spatially identify/differentiate tissue within the surgical field.	Resolution suitable to spatially identify/differentiate anatomical tissue with high anatomical detail.	T11 and T13–T17
**F9** Imaging rate	Video-rate imaging for instant surgeon feedback and seamless workflow integration.	Fast video-rate imaging for instant and smooth surgeon feedback and seamless workflow integration.	T19
**F10** Visualisation	Accurate visualisation of extracted information for surgical guidance.	Intuitive and accurate visualisation of extracted information for seamless surgical guidance.	T7 and T15–T18

**Table 2.** Overview of technical requirements of a hyperspectral imaging system for intraoperative surgical guidance. Corresponding functional requirements from table 1 are listed in the rightmost column.

	Minimum Requirement	Target Requirement	Req
**T1** System maintenance	System components may be effectively cleaned using a universal antimicrobial surface wipe.	Ibid. Additional camera housing resistance protects against dust and splashing liquids.	F1,F2,F4
**T2** Camera dimensions	Smaller than 10 × 10 × 12 cm^3^.	Smaller than 6 × 6 × 8 cm^3^.	F1,F2,F4,F5
**T3** Camera weight	Lighter than 1 kg.	Lighter than 0.5 kg.	F5
**T4** Camera housing	No sharp edges on camera housing.	Ibid.	F2,F5
**T5** Camera temperature	Temperature lower than 40 ^∘^C.	Ibid.	F2,F5
**T6** Camera connectivity	No more than two cables to provide power and fast data link	One cable to provide both power and fast data connection.	F2,F5
**T7** Light source energy	Adequate uniform coverage of required spectral range (cf T16).	Ibid.	F2,F3,F7,F10
**T8** Light source safety	Adherence to MPE limits with ionizing UV wavelengths ($&lt;400$ nm) eliminated.	Ibid.	F1,F2,F7,F10
**T9** System mount	Static system mount possible.	Adjustable system mount possible.	F1,F2
**T10** Camera settings	Manual adjustments of camera acquisition settings.	Automatic adjustments to obtain ideal camera acquisition settings.	F5,F6
**T11** Focus	Manual focus of target tissue.	Autofocus of target tissue.	F6–F8
**T12** Working distance	Fixed WD between 200 mm and 300 mm.	Variable WD between 200 mm and 750 mm.	F1,F3 and F5–F6
**T13** Field of View	Fixed FOV between 40 mm and 60 mm.	Variable FOV between 40 mm and 150 mm.	F6–F8
**T14** Depth of Field	At least 20 mm DOF for 50 mm FOV at fixed WD of 250 mm.	Variable 15 mm to 100 mm DOF.	F6–F8
**T15** Spectral bands	At least 16 spectral bands.	At least 100 spectral bands for fine spectral sampling.	F2,F7,F8,F10
**T16** Spectral range	At least a spectral coverage of 160 nm.	At least a spectral coverage of 500 nm.	F2,F7,F8,F10 and T7
**T17** Spatial image definition	1920 × 1080 pixels.	3840 × 2160 pixels.	F6–F8,F10
**T18** Image calibration	Satisfactory image calibration to enable reliable feature extraction during surgery.	Seamless and on-the-fly calibration possible for reliable feature extraction depending on surgical requirements and light conditions.	F3,F7,F10
**T19** Imaging rate	Video-rate imaging of at least seven FPS.	Video-rate imaging of at least 30 FPS.	F6,F7,F9

As part of surgical requirements, sterility of the iHSI system must be ensured so that safe handling by the surgical team is possible (F1), it must adhere to standard technical safety specifications (F2), light and illumination requirements must not impede surgical workflow (F3), and the device must be easy to maintain and clean in compliance with standard surgical practice (F4). It should be securely mounted during the procedure but the handheld device should be easily manoeuvrable, allowing for controlled mobilisation and immobilisation of the imaging system by a single operator without the need for an assistant (F5). The spatial resolution and spectral information captured within the surgical image must be compatible with the surgical action (F6), i.e. the provision of wide-field information covering the minimal region that provides sufficient context for surgical decision making. In addition, it should facilitate the ability of broader tissue surveillance relevant to the surgery. The device must be capable of providing critical functional or semantic tissue information and should be capable of providing detailed information on multiple features for comprehensive patient monitoring in order to increase surgical precision and patient safety during the procedure (F7). In the case of neuro-oncology surgery, this might be the demarcation of tissue boundaries to clearly demonstrate tumour tissue and its relation to critical brain structures such as nerves, blood vessels or normal brain. Furthermore, image resolution must be sufficiently detailed to facilitate spatial differentiation between tissue types within the surgical field of view F8). Imaging must be displayed at video-rate to facilitate instant surgeon feedback and seamless workflow integration with higher video-rates allowing for a smoother experience (F9). Accurate visualisation of extracted information is essential for surgical guidance whereby a better user experience can be achieved using intuitive display systems F10.

To ensure surgical safety and sterility, system maintenance should be straightforward and it should be possible to clean the system’s components effectively using a standard antimicrobial surface wipe (T1). The minimum requirements of HSI camera dimensions and weight are based on the estimates in Shapey *et al* (2018) obtained through a prototyping-testing design thinking methodology (Yock *et al* 2015), i.e. a camera smaller than 10 × 10 × 12 cm^3^ (T2) and lighter than 1.0 kg (T3). For a system with dimensions smaller than 6 × 6 × 8 cm^3^ standard drapes for covering the camera can be used to ensure sterility. Additionally, all camera edges must be smooth to prevent tearing of sterile drapes and injuring of staff members (T4). A maximum camera temperature of 40 ^∘^C ensures technical safety for device handling in addition to reduced dark currents for maintaining appropriate signal-to-noise ratios (SNRs) during image acquisition (T5). The number of cables for powering of and data connection with the camera must be kept at a minimum (T6). To enable adequate iHSI, a suitable light source must be available to provide sufficient energy across the active spectral range of the HSI camera (T7), but technical safety and light safety considerations must be adhered to so that no injury is caused to the patient due to light exposure (T8). This includes adhering to the maximal permissible exposure (MPE) with ionizing ultraviolet (UV) wavelengths below 400 nm (Yun and Kwok 2017). Light source setting adjustments must be possible to ensure optimal illuminant conditions for acquiring HSI information during surgery (F3,T10). Besides optimal light intensity settings depending on the surgical scene, this may include adjustment of optical filters to acquire high-fidelity HSI signal measurements depending on the imaging requirements of the HSI camera. Ideally, these settings are adjusted by automatically accounting for dynamic changes in the OR such as illumination. A static mounting system is the minimum requirement to ensure adequate intraoperative device handling (T9). Camera settings will need to be adjusted depending on the surgical context to acquire high-fidelity HSI information (T10). By meeting the target requirements for camera dimension and weight (T2,T3), further improvements in device handling may be achieved. High-fidelity tissue information requires the respective target tissue to be within the imaging field of view and kept in focus during HSI acquisition. During surgery this may require re-focusing which can either be achieved using manual or autofocus arrangements (T11). A fixed working distance (WD) between 200 and 300 mm (T12) with a fixed field of view (FOV) between 40 mm and 60 mm (T13) and a depth of field (DOF) of at least 20 mm (T14) are the minimum requirements necessary for iHSI (Nishiyama 2017) but the ideal scenario includes a system capable of variable WDs, FOVs and DOFs in order to maximise compatibility with current surgical visualisation systems (Langer *et al* 2020). The number of spectral bands, spectral range and spatial image definition largely depend on the clinical application to provide the critical functional and/or semantic features. Our assumption based on reviewing the previous literature and our own experience is that tens of well-defined spectral bands are required to achieve significant improvement with respect to standard RGB imaging. Based on the availability of industrial state-of-the-art snapshot HSI sensors (cf table 3), we specified that the minimum requirements for an iHSI system during surgery are 16 spectral bands (T15) and a spectral range of at least 160 nm (T16). Similarly, the use of at least 100 spectral bands with at least 500 nm spectral coverage is technically feasible (cf table 3), albeit at a lower frame rate, and likely to achieve superior tissue differentiation functionality. With the goal of providing information with at least 1 mm precision for reliable tissue differentiation during surgery, at least 3 pixels per millimetre are needed to visualise tissue boundaries. Following the minimum and target FOV requirements, imaging grids of at least 120 × 120 and 450 × 450 are therefore required. However, based on currently available HSI sensor technology, substantially higher resolutions are possible. Hence, we propose the resolutions of high-definition (1920 × 1080 pixels) and ultra high-definition (3840 × 2160) for minimum and target requirements, respectively (T17). Image calibration is crucial to obtain interpretable HSI data which typically includes the acquisition of both a white and dark reference image for white balancing to account for ambient light and specific camera settings (T18). This is typically achieved by acquiring images using a white reflectance tile and with a closed shutter, respectively. However, for surgical guidance in the OR, calibration data should ideally be available without having to interrupt the clinical workflow. The minimum imaging rate must be fast enough to provide real-time information suitable for surgical decision making without interfering with the surgical workflow (T19). Based on speed of processing in the human visual system an image visualisation rate faster than seven frames per second (FPS) is desired (Thorpe *et al* 1996). In some scenarios with a static scene, image acquisition rates of a few seconds per image per surgical scene may be sufficient to provide critical information to the surgical team. However, iHSI suitable for real-time image-guided surgery must be capable of providing video-rate imaging to ensure a live display of tissue information that is suitable also for dynamic scenes during surgery.

**Table 3.** Verification of intraoperative hyperspectral imaging systems based on whether requirements as outlined in table 2 are met. Assessment is performed for two camera setups with ratings (R) of 0 (minimum requirement not met), 1 (minimum requirement met) and 2 (target requirement met) using our current system designs.

	Linescan camera based	R	Snapshot camera based	R
**T1** Camera maintenance	All components may be effectively cleaned using a universal surface wipe.	1	Ibid.	1
**T2** Camera Dimensions	10 × 7 × 6.5 cm^3^.	1	6 × 6 × 5.4 cm^3^ (incl. heat sinks).	2
**T3** Camera Weight	0.58 kg.	1	0.28 kg (incl. heat sinks).	2
**T4** Camera housing	Camera housing with smooth edges.	2	Ibid. Additionally provided heat sinks with rounded edges.	2
**T5** Camera temperature	Active cooling system ensures low camera temperatures.	2	Passive cooling system (heat sinks) ensures low camera temperatures.	2
**T6** Camera connectivity	Two cables.	1	Single cable (GigE connection).	2
**T7** Light Source Energy	Xenon light source ensures sufficient illumination across VIS & NIR spectral ranges (250–1050 nm).	2	Ibid.	2
**T8** Light Source Safety	470–900 nm (VIS & NIR).	2	665–975 nm (NIR).	1
**T9** System mount	Not compatible with currently-available surgical supports.	0	Compatible with standard sterile mechanical arm systems.	2
**T10** Camera Settings	Adjustments possible using software control.	2	Ibid.	2
**T11** Focus	Optical system allows for manual focus for specific focal distance.	1	Ibid.	1
**T12** Working distance (WD)	System design using scope and adjustable lenses allows imaging distances between 250–750 mm.	2	Ibid.	2
**T13** Field of View	Optical system allows for 50 mm FOV at fixed WD of 250 mm.	1	Ibid.	1
**T14** Depth of Field	Optical system allows for 35 mm DOF for 50 mm FOV at fixed WD of 250 mm.	1	Ibid.	1
**T15** Spectral bands	150 + bands.	2	25 bands with 5 × 5 mosaic.	1
**T16** Spectral Range	470–900 nm (VIS & NIR).	2	665–975 nm (NIR)	1
**T17** Spatial Image Definition	3650 × 2048 pixels.	2	2048 × 1088 pixels with 5 × 5 mosaic, i.e. 409 × 217 pixels per band.	1
**T18** Image Calibration	Image calibration for specific camera/light settings based on white and dark reference images (dark reference is automatically acquired).	1	Image calibration for specific camera/light settings based on white and dark reference images.	1
**T19** Imaging Rate	2–40 s per image.	0	50 FPS.	2

### 2.2. Intraoperative HSI system design

By following the system design requirements above, we propose an iHSI system design as shown in figure 1. An HSI camera is connected to a sterile optical scope, such as a sterile exoscope, via an appropriate eye-piece adapter. Besides connecting the scope with the camera, such an adapter provides a means of adding spectral rejection filters and a control mechanism for zooming and focusing. The sterile optical scope is connected to the light source via a sterile light guide. Optical filters can also be placed in a filter wheel embedded in the light source to restrict the light source spectrum depending on the camera sensor or clinical requirements. The HSI camera is connected to a computational workstation via a connection that provides both power and a fast data link suitable for real-time HSI data transfer. The workstation processes the acquired HSI data for real-time visualisation of derived information. A sterile surgical drape, covering both the HSI camera and data cable, is sealed with the sterile exoscope ensuring sterility of the overall imaging system. Depending on the surgical application, the sterile imaging system may be hand-held by the operator or fixed to a surgical table using a standard mechanical arm permitting controlled mobilisation or immobilisation of the imaging system depending on the clinical requirements during surgery. By ensuring that the camera system is lightweight enough, its controlled mobilisation and immobilisation can be achieved by using a sterile or draped mechanical arm that attaches to the sterile optical scope. Such a mechanism allows positioning of the iHSI system at a safe distance outside the surgical cavity while the eye-piece adapter provides appropriate focusing capabilities for acquiring HSI data.

## 3. Experiments and results

We first present the specific system configuration that integrates two state-of-the-art industrial HSI cameras as part of our iHSI system setup. Both iHSI system setups are then evaluated and scored against the presented design requirements (tables 1 and 2). Following this, we perform a controlled checkerboard experiment to demonstrate that reliable reflectance measurements can be obtained with the proposed system using both HSI cameras. An *ex vivo* experiment is performed to investigate reflectance properties for a range of tissue types by building on a standard tripod system used for photography that allows for versatile imaging configurations in a controlled environment. Finally, we describe a successful ethically-approved in-patient clinical feasibility case study that demonstrates the ability of our real-time iHSI system to seamlessly integrate into the surgical workflow while respecting clinical requirements in the OR such as sterility.

### 3.1. HSI system configuration

Two hyperspectral imaging cameras were investigated as part of our proposed iHSI system (table 3): (a) a linescan HSI system using the Imec snapscan VNIR, i.e. visible (VIS) to near-infrared (NIR) region, camera and (b) a snapshot HSI system using the Photonfocus MV0-D2048x1088-C01-HS02-160-G2 camera.

The Imec system captures hypercube images with a spatial resolution of up to 3650 × 2048 pixels for 150 + spectral bands between 470 nm to 900 nm. The imaging speed to acquire a full hypercube ranges between 2 s and 40 s depending on acquisition parameters, illumination and imaging target. The camera without optics has a size of 10 × 7 × 6.5 cm^3^ and a weight of 0.58 kg. Imec’s linescan technology is characterised with high SNRs across the spectral range. An integrated shutter automatically measures dark currents therefore requiring only the manual acquisition of a white reference image for image calibration.

**Figure 2.**
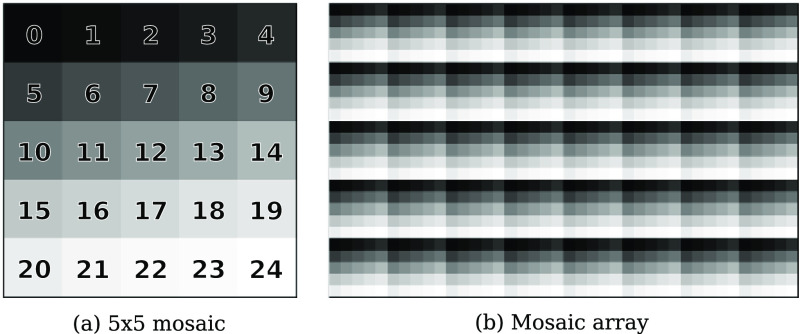
Illustration of the Imec snapshot mosaic CMV2K-SM5x5-NIR sensor which acquires 25 spectral bands between 665 and 975 nm in a 5 × 5 mosaic. Hyperspectral data is captured in a single shot (‘snapshot mosaic’) by acquiring spatially and spectrally interleaved information following a mosaic array arrangement.

The Photonfocus camera deploys the Imec snapshot mosaic CMV2K-SM5x5-NIR sensor which acquires 25 spectral bands in a 5 × 5 mosaic between the spectral range of 665 and 975 nm. With a sensor resolution of 2048 × 1088 pixels, hyperspectral data is acquired with a spatial resolution of 409 × 217 pixels per spectral band as illustrated in figure 2. Video-rate imaging of snapshot data is achieved with a speed of up to 50 FPS depending on acquisition parameters. The camera without optics has a size of 3 × 3 × 5.4 cm^3^ and a weight of 0.08 kg.

**Figure 3.**
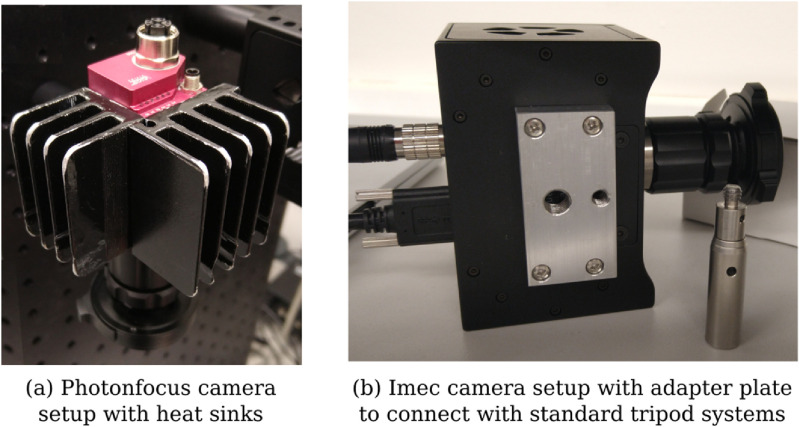
(a) Two custom heat sinks are mounted on the Photonfocus camera to keep operating temperatures low. (b) Custom adapter plates with 1/4-20 UNC and 3/8-16 UNC threaded holes were created for both Photonfocus and Imec cameras for use with standard tripod systems as described in section 3.4.

A passive prototype cooling system was fabricated with rounded edges and installed by mounting two heat sinks on the sides of the camera to keep operating temperatures, and therefore imaging noise, low during image acquisition (figure 3(a)). This increased the overall dimensions by about 3 cm in each direction with additional weight of about 0.2 kg.

An Asahi Spectra MAX-350 light source (300 W Xenon lamp) was used to provide broadband light. Depending on the experiment either a VIS module or UV-NIR mirror module was available which provided light over a 385–740 nm or 250–1050 nm region, respectively. In case of using the UV-NIR mirror module an additional 400 nm longpass filter (Asahi Spectra XUL0400) was placed in front of the mirror module to suppress ultraviolet (UV) light to improve the light safety profile. For the Photonfocus camera, a 670 nm longpass filter (Asahi Spectra XVL0670) was placed in the filter wheel to avoid signal contamination due to out-of-band sensor responses during image acquisition originating from sensor sensitivity to light in the VIS spectrum. Light intensity can be adjusted on the Asahi Light source between 5% and 100% using integer increments. The light source is connected via a Karl Storz fiber optic light cable 495NCS to a Karl Storz 0^∘^ VITOM surgical exoscope 20916025AA which allows imaging at a safe distance between 25 and 75 cm. A custom adapter was used to plug the light guide in the Asahi light source. The exoscope attaches to the respective HSI camera via individual RVA Synergies C-Mount 18–35 mm ZOOM Endoscope Couplers which additionally provide a manual zooming and focusing mechanism.

For calibration during all experiments, a 95% reflectance tile was used to acquire a white reference image. For the Photonfocus camera, a separate dark reference image was acquired with a cap to close the lens.

### 3.2. Verification of iHSI camera systems against design specifications

Both the linescan and snapshot camera-based iHSI systems were assessed towards the suitability for an intraoperative setup against the design requirements as specified in table 2. A summary of the assessment is provided in table 3.

**Figure 4.**
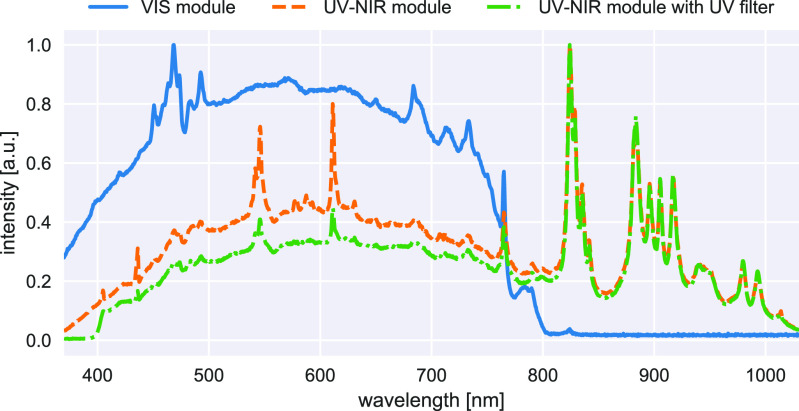
Measured spectrum of Asahi Spectra Xenon light source using the VIS and UV-NIR mirror modules. For the UV-NIR mirror module, the spectrum with and without additional UV filter was measured. Each curve was normalized based on maximum intensity.

Starting with the system requirements, sterility for both camera setups can be ensured using a combination of drapes and sterile components (T2). However, it is apparent from the respective camera specifications that the snapshot camera allows for a more compact iHSI system given its smaller camera dimensions and weight (T2,T3). The Xenon light source provides sufficient energy across VIS and NIR spectral ranges using the UV-NIR mirror module (T7) (250–1050 nm) as shown in figure 4. Light safety is ensured by blocking UV light using a 400 nm longpass filter (T8). The light source permits remote configuration using a serial communication protocol allowing for adjustment of filter wheel position and light intensity using customized software (T10). Similarly, both linescan and snapshot camera systems come with API interfaces to allow for remote control and software integration.

Device handling is critical to ensure camera systems can be mounted and moved securely during surgery without adversely impacting the surgical workflow and sterility (T2,T3,T10,T9). Due to the compactness of the snapshot camera-based system, this can be easily achieved using a mechanical arm construction (T9). However, for the linescan camera-based system, weight and form factor do not allow using the same approach. Mounting and pivoting in rotated positions of the camera system with weight supported only by the endoscope adapter and mechanical arm were not considered safe.

Both camera setups rely on the same optical setup and adapters and allow for imaging at a safe distance to the surgical cavity between 250 and 750 mm (T12). When using a fixed circular 50 mm FOV at a working distance of 250 mm both systems have a depth of field of 35 mm (T12,T13,T14) based on the exoscope manufacturer’s specification (Nishiyama 2017). Using the endoscope adapter, manual focus and zoom adjustments can be made to provide sharp imaging at a given focal distance (T11). In terms of HSI data quality, both spatial and spectral image resolution of the linescan camera is far superior than the snapshot camera (T15,T17). In particular, in addition to the fewer spectral bands sampled by the snapshot camera, additional postprocessing methods such as demosaicking, are needed to account for the sparse spatial sampling to obtain HSI data information on a sufficiently high spatial resolution for tissue analysis larger than 409 × 217 pixels per spectral band (T15,T17). While the linescan system covers a wide spectral range in both the VIS and NIR region to allow for rich feature extraction, the snapshot camera only provides NIR spectral information. Furthermore, the linescan technology comes with high-fidelity HSI signal measurement with high signal-to-noise ratios. In contrast, signals acquired using snapshot imaging are characterized by multimodal spectral band and crosstalk signal contamination resulting from the mosaic imaging sensor which needs to be accounted for. Consequently, the linescan system could potentially extract a wider range of relevant surgical features. However, the acquisition speed of the linescan camera between 2 and 40 s per image can interrupt the surgical workflow without providing video-rate information needed for real-time surgical guidance (T19). In particular, it is prone to motion artefacts if non-static imaging targets are imaged. In contrast, high frame rates of up to 50 FPS for the snapshot camera allow for real-time visualisation that can easily capture moving imaging targets (T19). For the linescan camera, image calibration can be achieved by acquiring a white reference image only due to its integrated shutter. For the snapshot camera, both a dark and white reference image needs to be acquired T18. For both camera setups, a robust calibration approach that can deal with changing illumination and imaging scenes is crucial to estimate reliable HSI information for intraoperative surgical guidance.

Overall, the linescan camera imaging quality is superior to the one provided by the snapshot camera. However, given its form factor, a more elaborate mounting mechanism needs to be designed to ensure safe and sterile handling of the camera during surgery. Moreover, its comparatively low imaging rate does not allow for HSI data capture without interrupting the surgical workflow which is crucial to provide real-time information for seamless surgical guidance. Nevertheless, its imaging characteristics can ensure high-quality HSI in controlled set ups. In contrast, the video-rate snapshot camera allows for a compact and sterile iHSI system that can be integrated into surgical workflows using standard clinical mechanical arm constructions. For reliable tissue analysis, image processing methods need to account for the reduced spatial and spectral image resolution in addition to lower signal quality that are characteristic for mosaic snapshot sensors.

### 3.3. Checkerboard study: iHSI system verification

**Figure 5.**
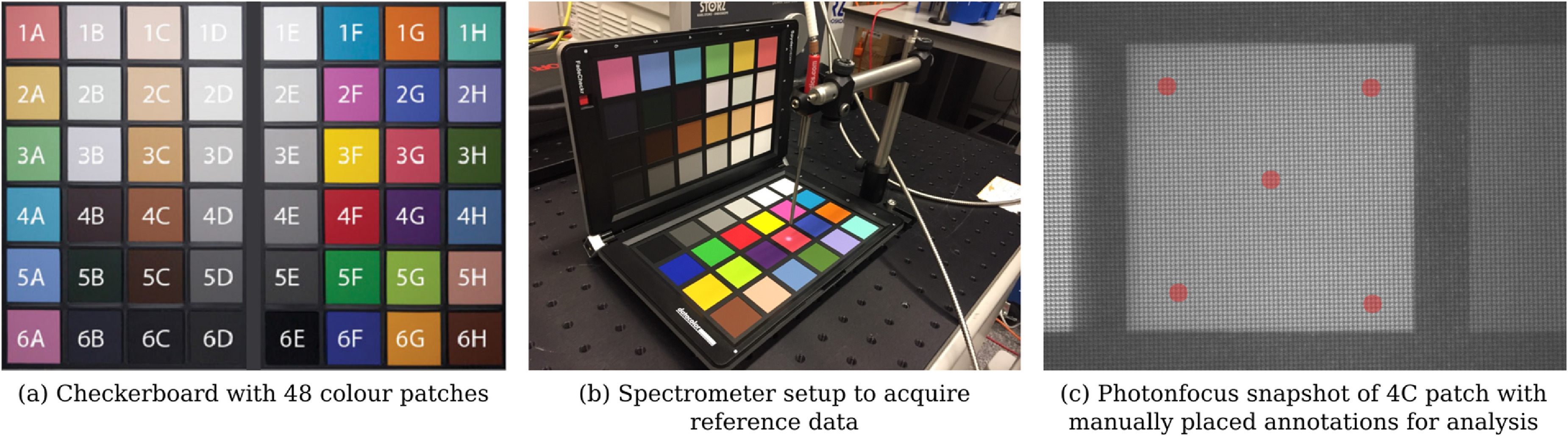
(a) Checkerboard with 48 colour patches used for HSI system validation. (b) Spectrometer setup to acquire reference data. (c) Snapshot mosaic image of 4C patch with five manually placed circular annotations of 10 pixel radius distributed over the patch for spectral analysis. The same annotation steps were performed to evaluate the respective linescan data.

Both the linescan and snapshot camera in section 3.1 were tested in combination with the proposed intraoperative optical system, i.e. the endoscope adapter and exoscope, to acquire HSI data in a controlled experiment using a datacolor SpyderCHECKR checkerboard which comes with 48 colour patches. For the experiments, the Asahi light source was used with the UV-NIR module in combination with the 400 nm longpass filter to provide light for 400–1050 nm. Reference spectra were acquired using an Ocean Optics Maya 2000 Pro 200–1100 nm spectrometer with an Ocean Optics QR600-7-VIS125BX reflectance probe (figure 5).

For the linescan camera, images were acquired using an exposure time of 10 ms and a gain of 1.2. For the snapshot camera, images were acquired using an exposure time of 15 ms and a gain of 2. Proprietary software was used to provide spectrally calibrated hypercube reflectance data for image analysis for both camera systems using the default image calibration files provided with the cameras as outlined in (Pichette *et al* 2017). In particular, given an acquired image $\mathbf{w}$ and white and dark reference images $\mathbf{w}_w$ and $\mathbf{w}_d$, the calibrated image is obtained by applying a correction matrix *C* to the white-balanced data $(\mathbf{w}-\mathbf{w}_d)/(\mathbf{w}_w-\mathbf{w}_d)$. Therefore, no dedicated system-wide calibration was performed to account for the specific light source intensity spectrum (figure 4) and individual optical components of the iHSI system—such as optical filters, endoscope adapter and exoscope—during image calibration.

Both linescan and snapshot camera systems were placed at a 35 cm distance to the checkerboard whereby images were acquired for each patch individually. For each calibrated hypercube image, five circular regions of 10 pixel radius, distributed over the colour patch, were manually segmented for spectral analysis (figure 5(c)).

**Figure 6.**
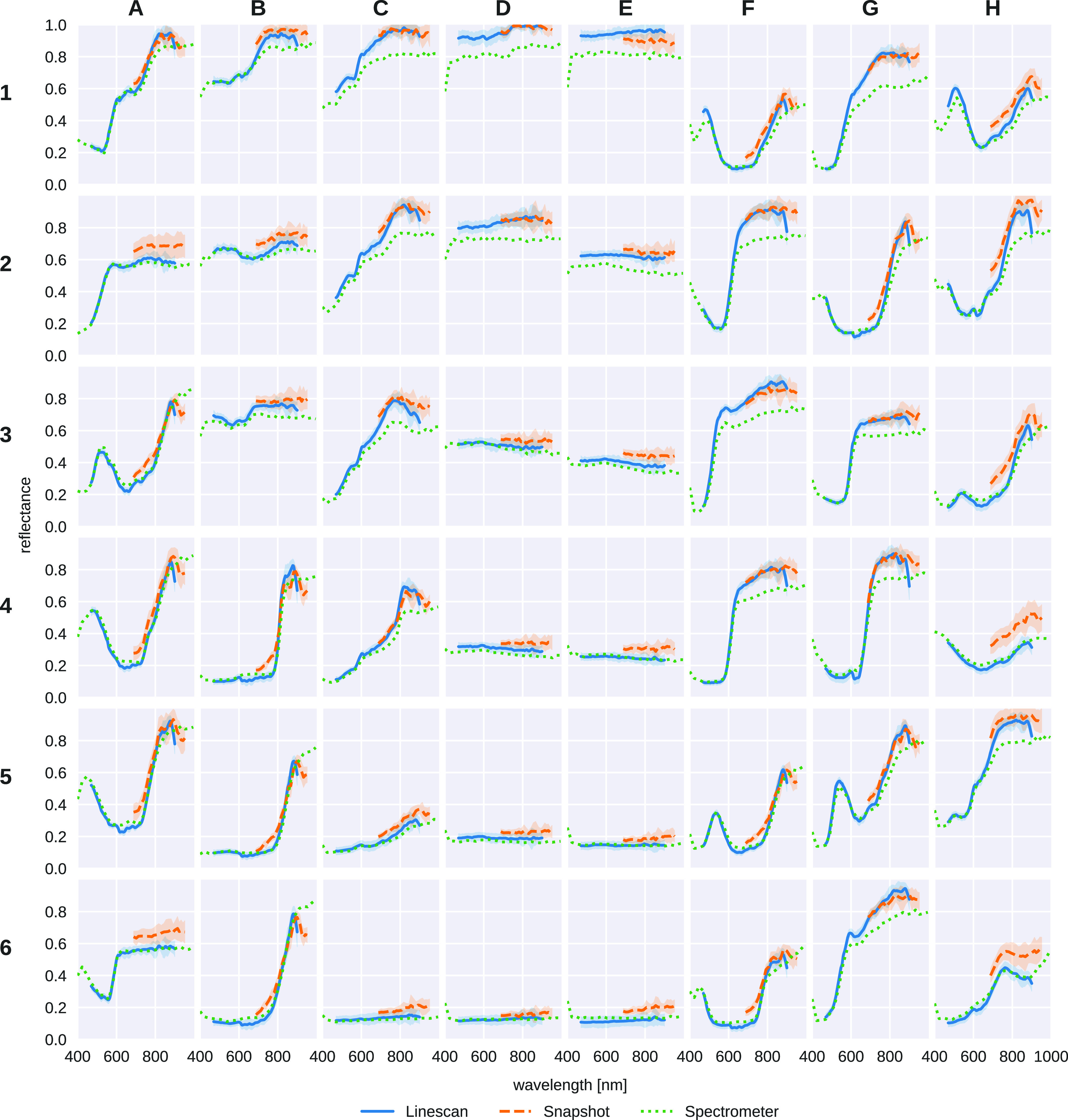
Comparison of measured reflectance curves between the linescan and snapshot iHSI camera systems and the reference spectrometer for each of the 48 colour patches. For both linescan and snapshot camera, mean and standard deviation of reflectance measurements within the manually segmented regions are shown.

Figure 6 provides a comparison between the reference data and spectral information obtained by the iHSI systems using the linescan and snapshot cameras. It can be seen that estimated reflectances for both linescan and snapshot iHSI systems largely follow the spectrometer reference measurements. However, especially for higher wavelengths, a sharp downward trend of estimated reflectances can be observed for a few patches (e.g. B4 and H2). Moreover, offsets for the snapshot system (e.g. A2 and A6) and for both the snapshot and linescan systems (e.g. D2 and G1) can be noticed.

### 3.4. Ex vivo study: cadaveric veal experiment

An *ex vivo* experiment using a fresh bovine calf cadaver was performed in a controlled environment to investigate tissue properties with the iHSI system setup for both linescan and snapshot cameras, performed at Balgrist University Hospital, Zurich, Switzerland. A bovine calf cadaver was selected because its anatomy approximates that of the human spine (Cotterill *et al* 1986).

**Figure 7.**
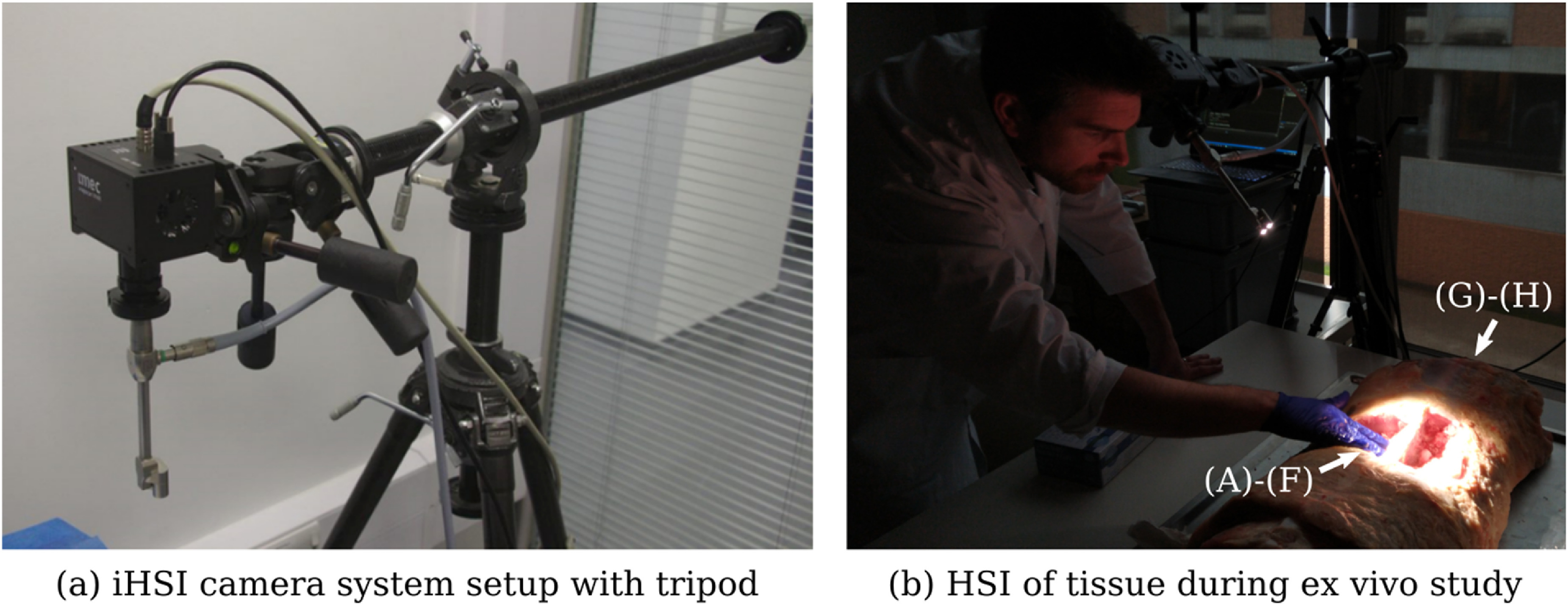
(a) Tripod setup with mounted intraoperative HSI (iHSI) system using the linescan camera for *ex vivo* experiments. A quick release plate used with standard tripod systems was used to mount linescan, snapshot and RGB cameras using custom adapter plates such as shown in figure 3(b). (b) Imaging setup during cadaveric veal experiments with orientated camera head for tissue assessment. Labels are provided to reference anatomical locations for tissue analysis shown in figure 9.

Various tissue types were exposed for tissue analysis including tendons, muscle, bone, joint capsule, dura and spinal cord. To achieve optimal orientation and position for imaging cadaveric tissue samples, a standard tripod system was used for mounting the iHSI camera systems (figure 7). For both linescan and snapshot cameras, secure attachment was achieved using custom adapter plates with 1/4-20 UNC and 3/8-16 UNC threaded holes (figure 3(b)). An additional Thorlabs DCC3260C RGB camera was used for the experiment to provide high-resolution 1936 × 1216 RGB imaging. The camera without optics has a size of 2.9 × 3.5 × 4.4 cm^3^ and a weight of 0.04 kg. Given its C-mount camera lens mount, it could be used with the same endoscope adapter as part of the same iHSI setup. Additionally, its housing comes with a 1/4-20 UNC threaded hole suitable for attaching quick release tripod plates.

**Figure 8.**
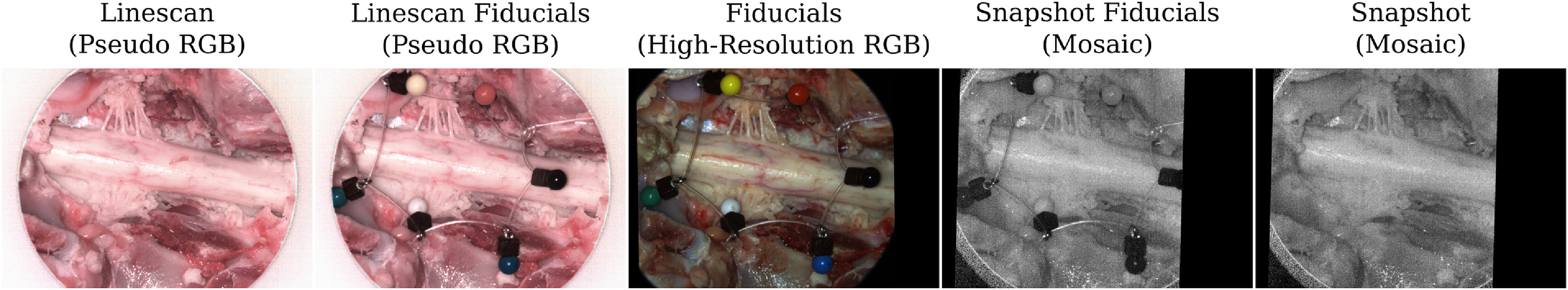
Example sequence of camera system acquisitions during *ex vivo* imaging to capture HSI data of the spinal cord and rootlets. With fiducials visible across the VIS and NIR spectrum, alignment between linescan (VIS & NIR) and snapshot (NIR) imagery was achieved using affine point-based registration for spectral analysis (figure 9).

Imaging of the exposed tissue using the three cameras followed the scheme as summarized in figure 8. By using separate fiducials which are visible and differentiable across the VIS and NIR spectrum it was ensured that images acquired with different cameras could be put in alignment retrospectively. We used a set of six pinheads with colours red, black, blue, white, green and yellow tied together with a nylon thread for facilitated handling during the experiment. After positioning the first HSI camera (either linescan or snapshot camera) and adjusting zoom and focus for imaging the tissue sample, fiducials were placed on the tissue to ensure they are within the FOV. Fiducials were then removed from the scene for image capture and carefully placed back to avoid anatomical changes before acquiring a second image with the same HSI camera. Without touching the scene, the HSI camera was swapped with the RGB camera using the tripod quick release mechanism to acquire an RGB image of the tissue sample with fiducials. Subsequently, without making changes to the scene, the RGB camera was swapped with the second HSI camera on the tripod. Minor adjustments to camera position, zoom and focus were typically needed to ensure the target tissue was in focus and the fiducials within the FOV before an image was acquired. After careful removal of the fiducials, another image was acquired of the same scene without making any other changes to the setup. For spectral analysis, a neurosurgeon (JS) manually annotated relevant tissue types in the pseudo-RGB linescan image, which was obtained by assigning the channels red, green and blue to the wavelengths of 660, 570 and 500 nm, respectively. By manually annotating the circular fiducials, alignment between all images was achieved using affine point-based registration (Myronenko and Song 2010). Manual segmentations in the linescan image space were then propagated to the snapshot image space for analysis using the obtained point-based affine registration.

During the *ex vivo* experiment, only the VIS mirror module was available for the light source therefore providing light between 385 and 740 nm. For NIR imaging with the snapshot camera, an additional 670 nm longpass optical filter in the filter wheel of the light source was activated. A gain of 3.01 and exposure time of 20 ms were used for the snapshot camera for all scenes whereby video imaging was performed to acquire multiple images of each individual static scene. On average, this led to the acquisition of 18 snapshot mosaic images per scene whose mean image was used for spectral analysis. For the linescan camera, a gain of 2 and exposure time of 20 ms were used. Light intensities were set to 100%, 100% and 50% for the snapshot, linescan and high-resolution RGB cameras, respectively. Imaging for all cameras was performed with room lights switched off and window blinds down to reduce the impact of background light. To ease the imaging workflow, acquisition of reference data for image calibration was performed once for both linescan and snapshot cameras in the beginning and the end of the experiment, respectively. Therefore, the same white balancing information for each HSI camera was used for data calibration of all imagery associated with different anatomical locations.

**Figure 9.**
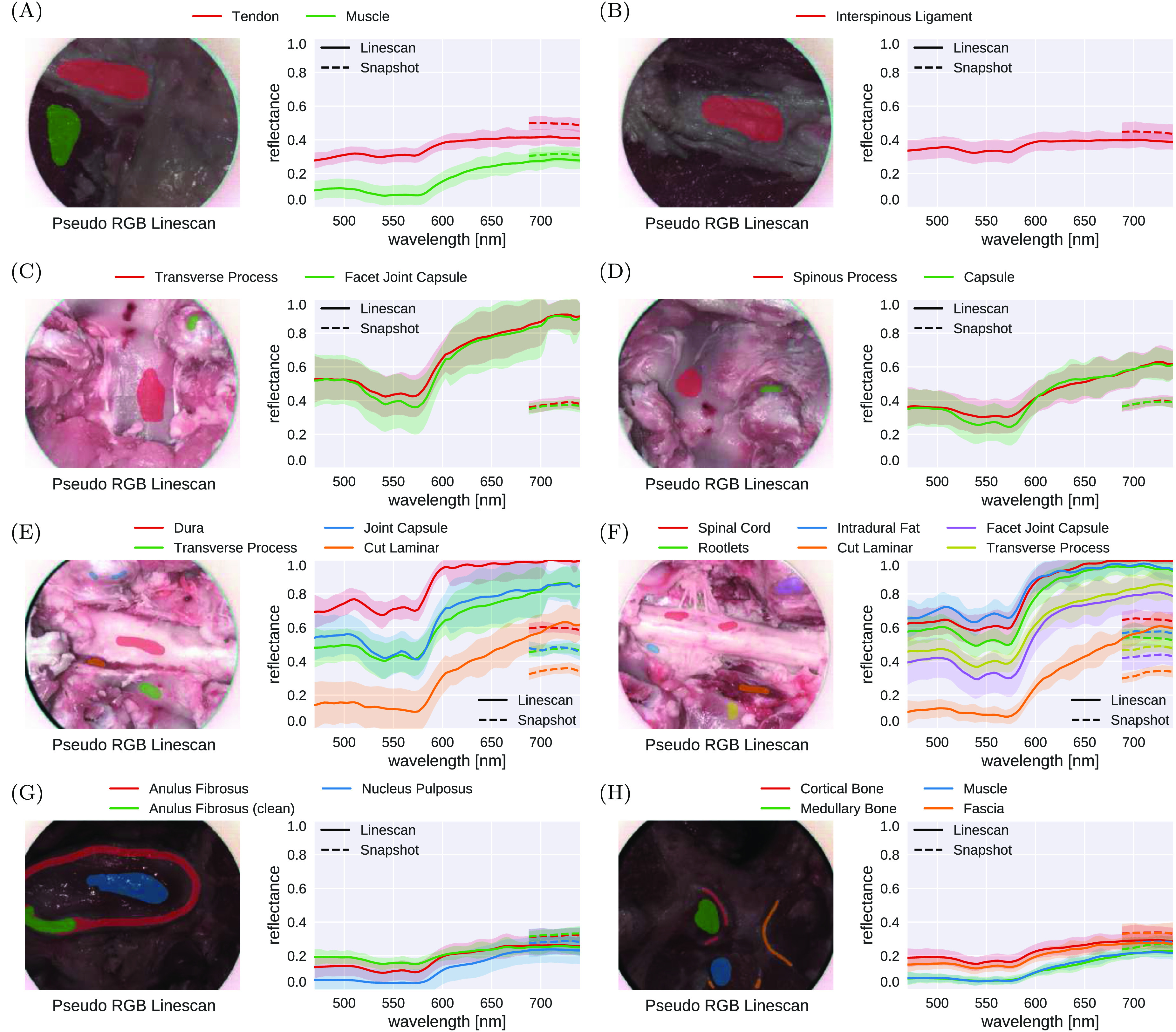
Comparison of reflectance curve measurements between 470 and 740 nm associated with *ex vivo* tissue sample imaging of eight different anatomical scenes shown in figure 7(b) using the proposed iHSI system with linescan and snapshot cameras. For each camera, both the mean and standard deviation of reflectance measurements within the manually segmented regions are shown. Relative distribution and qualitative behaviour of reflectance values across tissue types between the cameras are well aligned. However, quantitative measurements of tissue reflectances between cameras generally deviate from each other likely due to different white balancing requirements associated with imaging multiple anatomical regions during the experiment.

Figure 9 provides a comparison of estimated reflectance curves between 470 and 740 nm of both linescan and snapshot-based iHSI systems for eight different anatomical scenes, referenced in figure 7(b). For the snapshot camera, only 5 out of 23 reconstructed bands were available for analysing the measurements between 670 and 740 nm. In general, relative distribution and qualitative behaviour of reflectance values across tissue types for overlapping spectral bands between the cameras are well aligned. However, it can be observed that quantitative measurements of tissue reflectances between cameras tend to deviate from each other likely due to non-uniform white balancing requirements for imaging different anatomical sites. In particular, changes in position and angulation of the camera head needed to capture data of different anatomical sites have likely resulted in different lighting conditions and therefore white-balancing requirements which was not adequately compensated for using a single pair of reference images.

### 3.5. In patient clinical feasibility case study: spinal fusion surgery

Following the assessment of the proposed iHSI system for both linescan and snapshot cameras against design requirements critical for surgery in section 3.2 in combination with the quantitative and qualitative assessments in sections 3.3 and 3.4, the snapshot-based system appears suitable for providing real-time HSI that can seamlessly integrate into the surgical workflow. To test this hypothesis, we conducted an intraoperative clinical feasibility single-patient case study as part of a spinal fusion surgery at Balgrist University Hospital, Zurich, Switzerland. The study was approved by the cantonal ethical committee (BASEC Nr: req-2019-00939).

Figure 1 presents a schematic of the iHSI setup deployed in surgery. In addition to the system components described in section 3.1 a standard Karl Storz mechanical arm was used for safe attachment of the iHSI camera system to the surgical table using an articulated L-shaped stand (28272HC) via a clamping jaw (28272UGK). Safe attachment to the snapshot HSI camera via the VITOM exoscope was achieved via an appropriate rotation socket (28172HR) and a clamping cylinder (28272CN). Overall sterility of the system was ensured by autoclaving the mechanical arm, the exoscope and the light guide before surgery and draping the camera and associated cable.

**Figure 10.**
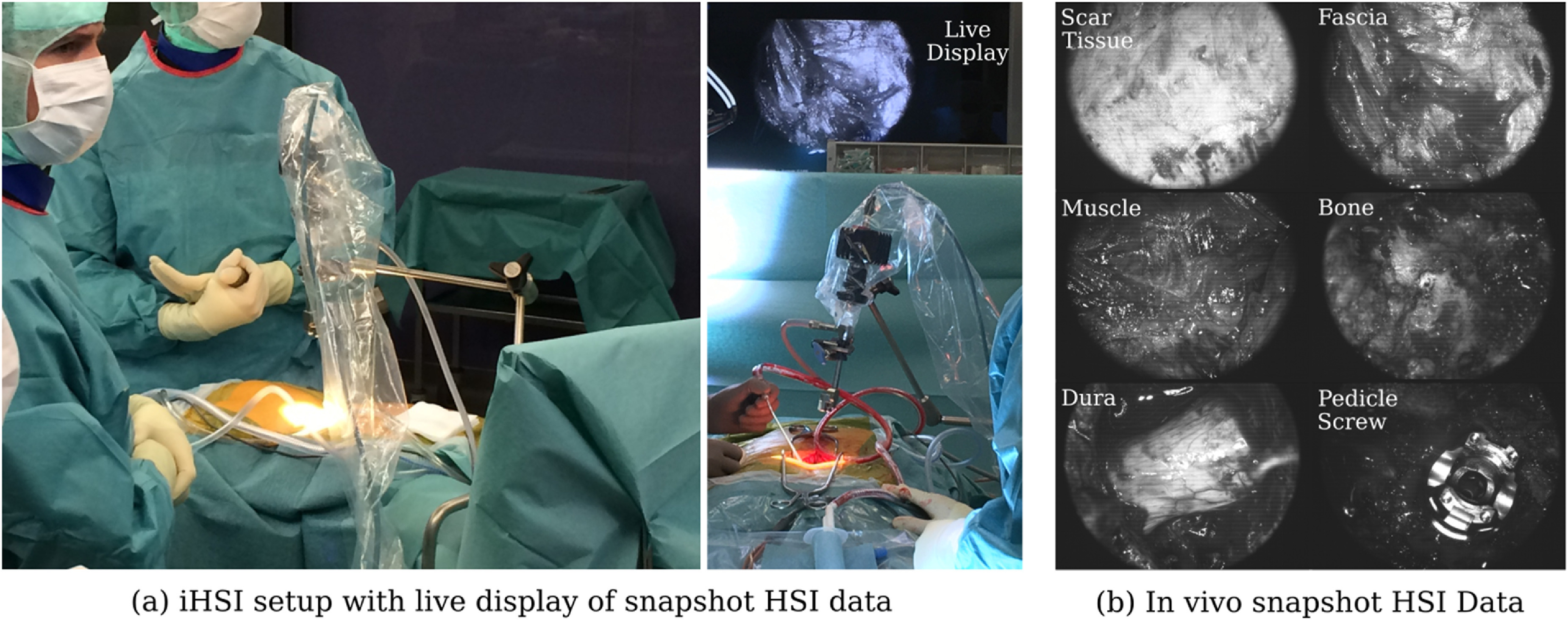
(a) Intraoperative HSI (iHSI) setup during spinal fusion surgery. A live display provides real-time visualisation of snapshot HSI data. (b) Example snapshot mosaic images of acquired *in vivo* HSI imagery.

The primary goal of the intraoperative clinical feasibility case study was to assess the system’s integration into the standard surgical workflow. To focus on this objective, we chose to mimic current optical camera systems during surgery and used white light between 385 and 740 nm without the 670 nm longpass filter^12^ 12While theoretically possible to obtain quantitative results for up to five bands of the NIR camera with the available VIS light source, the requirement of the 670 nm longpass filter would have resulted in unconventional, and for the surgical team potentially distracting, red light during surgery. Given the limited value of, at most, five spectral bands and the desire to minimize the risk profile for the patient during surgery, we decided to perform the workflow study using familiar white light.. For the snapshot camera, a gain of 4 and exposure time of 20 ms was chosen with the light source providing 100% of light intensity. A laptop running customized software for real-time interaction with the camera system and data visualisation was placed onto a trolley at a safe distance outside of the sterile environment. Connection to a monitor in the OR provided a live display of captured video-rate HSI data (figure 10(a)). In particular, this allowed for instant feedback to and interaction with the surgical team for adjusting camera position and orientation in addition to endoscope adapter settings to acquire in-focus data for the region of surgical interest. Using this setup, *in vivo* imaging was performed at eight different stages during surgery to acquire HSI data of various tissue types including skin, adipose tissue, scar tissue, fascia, muscle, bone, pedicle screws and dura (figure 10(b)). Imaging of each anatomy lasted between 6 s and 44 s with minimal disruption to the surgical workflow. After successful surgery with seamless transitions to acquire HSI data, a final recording of 3 min 16 s was performed to capture imaging data covering the surgical cavity.

## 4. Discussion and conclusion

Previous work has underlined the potential of HSI for intraoperative tissue characterisation as a non-contact, non-ionising, non-invasive and label-free imaging modality. Despite numerous research studies exploring the clinical potential of HSI for surgery, to our knowledge, no HSI system has been presented which follows strict clinical requirements including sterility and seamless integration into the surgical workflow providing capabilities to deliver real-time information with potential for intraoperative surgical guidance.

Towards reaching this goal, here we present system design requirements of an HSI system critical for intraoperative surgical guidance suitable for open surgery. However, given the design of the system, straightforward adaptation for endoscopic surgery is possible. We investigate two state-of-the-art industrial HSI camera systems, based on either linescan or snapshot technology, and assess their suitability for surgical use. Based on our established criteria, we present an intraoperative HSI system and perform a scoring against these requirements by considering both HSI cameras. We performed controlled checkerboard experiments demonstrating that reliable reflectance measurements can be obtained with the proposed system using both HSI cameras. However, quantitative experiments underline that a more refined calibration model needs to be deployed that accurately describes the intraoperative optical system for more precise reflectance measurements. A series of *ex vivo* experiments were performed to investigate reflectance properties for a range of tissue types including tendons, muscle, bone, joint capsule, dura and spinal cord with both iHSI camera setups mounted on a standard tripod system allowing for versatile imaging configurations in a controlled environment. In particular, this proved to be a suitable setup for the linescan camera to provide high-resolution data in both spatial and spectral dimensions across the VIS and NIR spectrum for *ex vivo* tissue analysis. The iHSI system allowed for seamless and safe transitions during various stages of spinal fusion surgery and acquired video-rate HSI data of multiple tissue types including skin, adipose tissue, fascia, muscle, bone, pedicle screws and dura. Our successful clinical feasibility case study demonstrated that the proposed iHSI system seamlessly integrates into the surgical workflow by respecting critical clinical requirements such as sterility and is capable of providing wide-field video-rate HSI imagery. By developing a data-driven information processing pipeline we believe such video-rate HSI data can be utilised to provide real-time wide-field tissue characterisation for intraoperative surgical guidance.

Because of technical limitations and consideration not to alter the view of the surgeon, only visible light between 385 and 740 nm was available for use in the *ex vivo* and *in vivo* studies at Balgrist University Hospital. Given that the snapshot NIR camera operates between 665 and 975 nm only 5 out of 25 acquired spectral bands could be used for image analysis. Computational simulations not presented in this study suggested that these five reconstructed bands can still provide accurate reflectance information but may be less reliable for higher wavelengths within this range. Furthermore, only the default calibration files associated with each camera were used for this study to reconstruct reflectance data using the proprietary software. In particular, the specific spectrum of the Xenon light source was not taken into consideration. Given the stark non-uniformities especially in the NIR region greater than 800 nm (figure 4) inaccuracies are to be expected. In addition, spatial changes in illumination due to, e.g. vignetting and optical changes induced by different zoom and focus settings of the endoscope adapter were not taken into account. Moreover, despite demonstrating the suitability of our proposed tripod-based iHSI system setup for *ex vivo* tissue analysis experiments, the quantitative reflectance measurements presented as part of this study are likely to be confounded due to imprecise white balancing. In particular, this illustrates the difficulty to acquire reliable calibration data in practice to acquire quantitative HSI data for different imaging scenarios, especially during surgery. However, qualitative behaviour of reflectance measurements captured for multiple tissue types may still persist due to good qualitative correlation between radiance measurements and calibrated reflectance measurements.

Future work includes further characterising the optical components of the iHSI setup to achieve more accurate calibration models. In particular, this includes the optical subsystem consisting of exoscope and endoscope adapter for different zoom and focus settings. Moreover, further computational algorithms will be developed to reconstruct real-time hypercube data from spatially and spectrally undersampled characteristic for snapshot mosaic imaging. Building on the NIR snapshot camera, this can be used to provide real-time information on blood perfusion and oxygenation saturation which can help, e.g., to differentiate healthy from necrotised tissue during gastrointestinal surgery. The proposed *ex vivo* setup can be used for further experiments to acquire both high-resolution linescan and low-resolution snapshot HSI data. This can provide crucial information for developing real-time demosaicking and tissue differentiation methods for snapshot HSI.

Additional experiments should be based on a snapshot system that operates in the VIS range instead. Indeed, given our setup, any compact camera that follows camera dimension and weight requirements as outlined in table 1 can be integrated into the proposed iHSI system setup in a straightforward manner. It would also be beneficial to enable HSI acquisition with a variable working distance, field of view, depth of field and depth of focus (table 2) which in turn would enable the device to be integrated with various commercial exoscopic surgical systems (Langer *et al* 2020) and would ensure parity with current microscopic standards (Schwiegerling *et al* 2015). Further experiments will be performed to investigate the accuracy at which tissue margins can be identified. While at least 3 pixels per millimetre are a necessary requirement to provide at least 1 mm precision for margin differentiation, the ability to resolve tissue boundaries also depends on light diffusion effects in tissue which may additionally vary in between different organs and anatomies.

Our *in vivo* clinical feasibility case study demonstrated that our iHSI device integrated well into a standard surgical workflow and was capable of capturing HSI data. Overall, members of the surgical and theatre team found the system straightforward to use although routine training will need to be implemented to ensure smooth operating during surgery. The current hardware setup and draping requirements posed no safety concerns to team members and the system’s size, weight and portability were acceptable in maintaining a smooth surgical workflow. Some team members commented that having to dim the lights to acquire HSI was a little limiting. Further work to provide on-the-fly image calibration crucial to acquire interpretable and quantitative HSI data that can deal with changing light conditions in the OR during surgical procedures such as recently proposed in Ayala *et al* (2020) is therefore in progress. Additionally, we intend to conduct further in-patient intraoperative studies with a larger number of patients to fully assess and appraise the use of our iHSI device during surgery.

## Data Availability

The data that support the findings of this study are available upon reasonable request from the authors.
